# Pleomorphic Carcinoma Arising at Previous Resection Margins of Ground-Glass Opacities

**DOI:** 10.1016/j.atssr.2023.12.025

**Published:** 2024-02-08

**Authors:** Tomoaki Kinno, Toshiro Futagawa, Kenji Suzuki

**Affiliations:** 1 Department of General Thoracic Surgery, Juntendo Tokyo Koto Geriatric Medical Center, Tokyo, Japan; 2 Department of General Thoracic Surgery, Juntendo University School of Medicine, Tokyo, Japan

## Abstract

Pleomorphic carcinoma of the lung is highly malignant and occurs mostly in the upper lobes of smokers; however, no reports exist of new occurrences from the resection margins of the previous ground-glass opacities. In our case, a 73-year-old man underwent an initial partial resection of the left upper lung for ground-glass opacities, with progressive thickening of the resection margin during the following 10 months. Radical resection was performed, resulting in the diagnosis of a newly arising pleomorphic carcinoma from previously resected margins.

Pleomorphic carcinoma of the lung has a poor prognosis. It commonly occurs in heavy smokers and affects the peripheral lungs. However, the carcinoma is rarely diagnosed preoperatively because of the low frequency. Tumors arising from resected lung cancer margins are often recurrent or present as granulomas. In this report, we describe a case in which after resection of both lungs for multiple ground-glass opacities, a new-onset pleomorphic carcinoma arising at the previous left lung resection margins was diagnosed through surgical intervention.

A 73-year-old man had a history of diabetes, heavy smoking, and alcohol consumption. He was diagnosed with multiple ground-glass opacities and underwent partial resection of the left lung by video-assisted thoracoscopic operation. Then, after 3 months, he underwent a right upper lobectomy by video-assisted thoracoscopic operation. On pathologic examination of bilateral ground-glass opacities, 3 left lung lesions (1 minimally invasive adenocarcinoma Figure 2A, and 2 adenocarcinomas in situ) and 2 right lung lesions (2 minimally invasive adenocarcinomas), with no cancerous remnants at each resection margin, were identified. At 10 months after the initial left lung operation, the left lung resection margin had progressively thickened and displayed a high positron emission tomography (PET) accumulation, with a maximum standardized uptake value of 9.2 (Figure 1). Diagnostic resection by open thoracotomy was proposed for suspected recurrence or new malignant transformation. Surgical findings demonstrated strong adhesions around the surgical wound. Most of the tumor was necrotic, and invasion into the aorta was suspected; however, intraoperative rapid diagnosis of the tumor and the margin was negative for malignancy. Complete resection was achieved by left lung S1+2 segmentectomy and hilar nodal sampling. On pathologic examination, gross findings displayed that the tumor was milky and viscous with a coarse structure. On microscopic examination, numerous polymorphous atypical cells were present along the resection margins without stromal components (Figure 2B). Immunostaining revealed that the atypical cells were positive for cytokeratin, MIB1 (>30% positive), and vimentin (Figures 2C-2E) and negative for TTF1 and p40, indicating a pleomorphic carcinoma T4 N0 M0 stage IIIA arising at the previous dissection site. The tumor had a mesenchymal-epithelial transition factor (MET) exon 14 skipping mutation and was strongly positive for programmed cell death ligand 1. The perioperative course was uneventful. The patient refused to undergo postoperative adjuvant therapy. Furthermore, he is currently alive at 2 years and 11 months postoperatively, with no recurrence.

**Figure 1:**
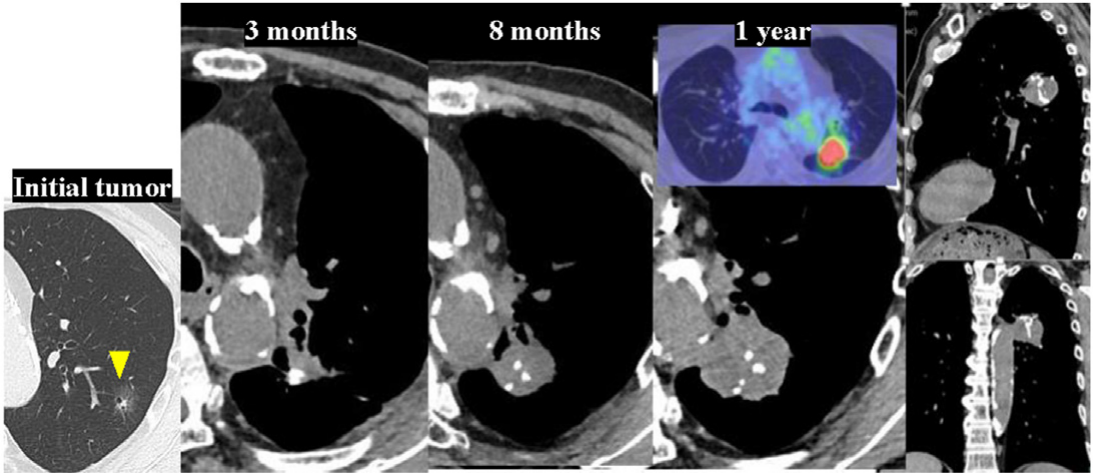
Temporal changes on chest computed tomography of the initial tumor (arrowhead) and the associated resected margins are demonstrated. The tumor at the resection margin is enlarged to a maximum diameter of 35 × 32 mm, and the maximum standardized uptake value on ^18^F-fluorodeoxyglucose positron emission tomography is 9.2.

**Figure 2:**
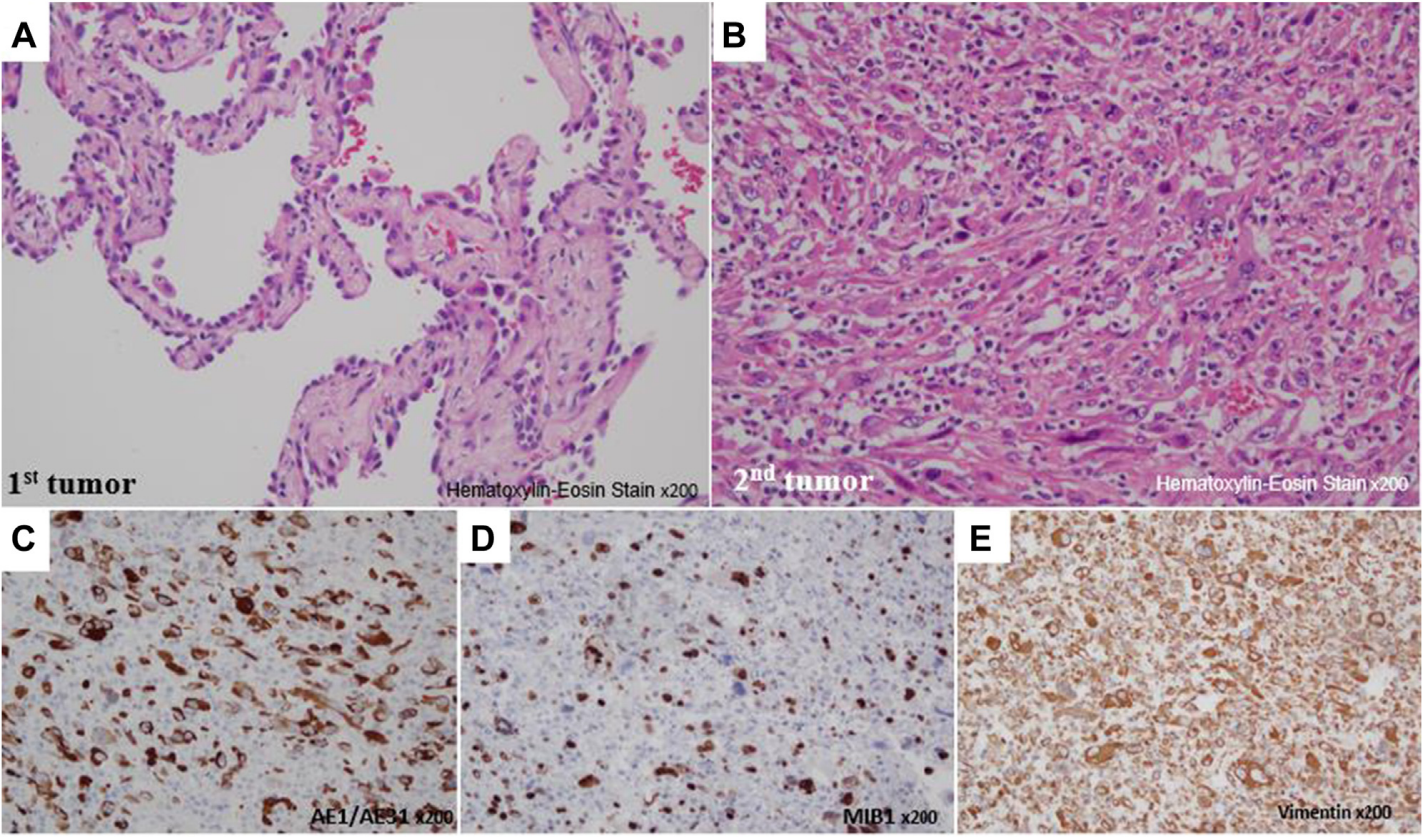
(A) The pathologic examination of the first surgical left lung ground-glass opacities (minimally invasive adenocarcinoma) is displayed, and (B) it is a newly arising pleomorphic carcinoma, with hyperplastic atypical cells and mitotic nuclear images (hematoxylin and eosin, magnification ×200). The atypical cells are positive for (C) mixture of 2 different clones of anticytokeratin monoclonal antibodies (AE1 and AE3) (D) E3 ubiquitin ligase mind bomb 1 (MIB1) (>30% positive), and (E) vimentin and negative transcription termination factor 1 (TTF1), novel aspartic proteinase of the pepsin family (Napsin A), p40, and p53, suggesting pleomorphic carcinoma.

## Comment

Primary pleomorphic carcinoma of the lung is a histologic type proposed in the 2001 World Health Organization classification and is also defined in the eighth edition of the American Joint Commission on Cancer TNM staging system. The tumor has a low frequency of 0.3% and tends to be more common in heavy smokers, in the right upper lobe, and in the male sex.^1^ Pleomorphic carcinoma grows rapidly, with an average tumor doubling time of 30 to 52 days,^2^ reflecting poor prognosis and refractory nature. Although surgery has recently been the mainstay of treatment, some patients respond to immunotherapy, resulting in prolonged survival.

Tumors arising at the lung resection margins are of concern for cancer recurrence or benign tumors, such as granulomas and non–tuberculosis mycobacteria, which are difficult to diagnose preoperatively. In addition, PET can be used to evaluate lung cancer; pleomorphic carcinomas exhibit a higher maximum standardized uptake value than the other histologic types.^1^^,^^2^ Granulomas and infectious lesions can also display high accumulation, making differentiation between them and cancer difficult. In our case, as the thickening of the margins progressed rapidly on computed tomography (CT), we initially assumed a latent or benign tumor because the previous lung cancer was a histologically low-grade adenocarcinoma with negative surgical margins. Based on the tumor’s increased growth rate and high PET accumulation, we argued for diagnostic operation, which resulted in the identification of a new pleomorphic carcinoma.

The diagnosis of tumors arising at previous lung resection margins is variable, and little knowledge of specific differential diagnostic methods or devices is present. In CT diagnosis, there are reports of differential diagnosis based on the comparison of CT values, growth speed (≥2 mm),^3^ and shape of the thickness (concentric or radial).^4^ Mizuno and coworkers^5^ combined multiple findings, such as a short disease-free interval, high carcinoembryonic antigen level, and PET value of ≥2. Although a few reports mentioned genetic mutations in cut-end second tumors, Isaka and colleagues^6^ summarized 6 cases of cut-end second tumors after resection of adenocarcinoma in situ and mentioned the epidermal growth factor receptor (EGFR) expression status and treatment methods for both primary and secondary tumors.

In terms of the pathophysiology of the development of pleomorphic carcinoma, the patient’s background is unique, with a high incidence of smokers and patients with diabetes; some cases occur in an inflammatory background, such as pyothorax or with EGFR/MET mutation.^1^ Furthermore, as an effect of chemotherapy, Inoue and colleagues^7^ reported a case of pleomorphic carcinoma after cytotoxic chemotherapy that followed operation for ground-glass opacities, and reports of transformation to pleomorphic carcinoma after EGFR–tyrosine kinase inhibitor treatment are available.^8^ In our pleomorphic case, the effect of surgical stimulus on cancer development was not clear; however, the presence of the MET mutation may indicate that some changes occurred during the process. We hope that the accumulation of cases will help elucidate the mechanism of carcinogenesis in the future.

In conclusion, we encountered a case of new-onset pleomorphic carcinoma arising at the previous resection margins of ground-glass opacities. In cases with rapid thickening of resection margins and strong PET accumulation, the diagnosis should include the possibility of pleomorphic carcinoma, considering the patient's background.
